# Metatranscriptomic dynamics after *Verticillium dahliae* infection and root damage in *Olea europaea*

**DOI:** 10.1186/s12870-019-2185-0

**Published:** 2020-02-17

**Authors:** Jose Manuel Martí, Luis F. Arias-Giraldo, Wladimiro Díaz-Villanueva, Vicente Arnau, Antonio Rodríguez-Franco, Carlos P. Garay

**Affiliations:** 1 Biological Systems and Engineering Division, Lawrence Berkeley National Laboratory, 1 Cyclotron Rd, Berkeley, CA, 94720 USA; 2 DOE Joint BioEnergy Institute, 5885 Hollis St, Emeryville, CA, 94608 USA; 3 Institute for Integrative Systems Biology (I2SysBio), UVEG-CSIC, Catedrático José Beltrán, 2, Paternas, Valencia, 46980 Spain; 4 Institute for Sustainable Agriculture - CSIC, Avenida Menéndez Pidal s/n, Córdoba, 14004 Spain; 5 Department of Biochemistry and Molecular Biology, University of Cordoba, Campus de Rabanales, Edificio Severo Ochoa, Córdoba, 14071 Spain; 6 Laboratorio Subterráneo de Canfranc (LSC), Camino de los Ayerbes s/n, Canfranc-Estación, Huesca, 22880 Spain

**Keywords:** Temporal metatranscriptomics, Polymicrobial infection, Verticillium wilt of olive, *Olea europaea*, *Verticillium dahliae*, Biotroph, Necrotroph, Hemibiotroph, Root damage, Endophytes, Rank dynamics

## Abstract

**Background:**

The olive tree is of particular economic interest in the Mediterranean basin. Researchers have conducted several studies on one of the most devastating disorders affecting this tree, the Verticillium wilt, which causes substantial economic losses in numerous areas. We analyzed metatranscriptomic samples taken from a previous study conducted on leaves and roots of *Olea europaea* that were infected with *Verticillium dahliae*. In addition, we also analyzed mechanically damaged roots. The aim of our approach is to describe the dynamics of the root microbiome after severe perturbations.

**Results:**

Our results not only describe the dynamics of the microbial community associated with the disturbance, but also show the high complexity of these systems and explain how this can lead to a conflicting assignment of the various types of parasitism observed in a specific organism.

**Conclusions:**

Our findings indicate that this infection, although led by *Verticillium*, is driven not by a single species, but by a polymicrobial consortium that also includes natural endophytes of the olive tree. This community contains both biotrophic and necrotrophic organisms that alternate and live together during the infection. In addition, opportunistic organisms appear that take profit not from plant tissues, but from new emerging populations of microorganisms. Therefore, this system can be described as a complex biological system composed of different interacting communities. Notably, our work has important considerations when it comes to classifying the type of parasitism of a given species.

## Background

The olive tree could be the earliest cultivated temperate fruit since paleobotanists have traced back its domestication to the early Neolithic age [1]. At present, both the cultivation of olive and the olive-oil related industry have grown to the point of having a profound socioeconomic and environmental impact worldwide [2].

The Verticillium wilt of olive is one of the most devastating disorders affecting this crop. A recent transcriptomic RNA-seq analysis studied the interaction between *Olea europaea* and *Verticillium dahliae* [3], and concluded that mainly a ROS (reactive oxygen species) response appeared first in the pathogen and later in the plant. In this study, authors characterized differential expression of poly-A enriched mRNA in roots 2 and 7 days after the infection. However, the public SRA database containing these data, also includes reads after 15 days of infection that were not analyzed or taken into consideration in the above study.

In recent years, there has been a deviation from studying individual microorganism to study the entire community that is actually living in a particular niche. High-throughput nucleic acid sequencing, like shotgun metagenomics and metatranscriptomics (MTS), provides the additional advantage of not requiring prior knowledge of the organisms that are present in the analyzed environment.

Soil constitute one of the most complex systems in nature, where many different life forms interact with each other. Fungi play a major role as they act as parasites, saprotrophs, or mutualists in a myriad of environments, including the rhizosphere of many different plants [4]. The description of these interactions are, nevertheless, variable and somehow confusing in many cases. So, *V. dahliae* has been described as an hemi-biotrophic pathogen because it seems to behave as biotrophic during the initial stages of a plant infection, but changes to a necrotrophic lifestyle during subsequent stages [5–7]. The same goes for other fungi, such as those belonging to the genus *Fusarium* [8, 9]. However, the molecular or biological bases that underlie these different parasitical alternations are not yet fully understood.

The ENA *Mgnify* microbiome database contains many studies and analyses related to the fungal population composition associated with plants. The number of cases dealing with a fungal plant infection are however very scarce in this database. So, *Mgnify ID*MGYS00001376 refers to the still unpublished study of the infection of pedunculate oak by *Erysiphe alphitoides*, the causal agent of oak powdery mildew. And *Mgnify ID*MGYS00002393 applies to the temporal dynamics of bacterial and fungal communities during the infection of *Brassica rapa* roots by the protist *Plasmodiophora brassicae* [10]. Some recent works [11] focus on the characterization of fungal metagenomics in animal species, especially those from pig and mouse microbiomes. Human metagenomics studies, particularly those related to gut microbiome, have experienced a dramatic rise, mainly due to unexpected consequences for health and disease [12], which are especially evident through temporal dynamics analysis [13]. However, generally speaking, the composition and dynamics of the microbiome of animals, plants and fungi are not sufficiently studied compared to the human microbiota.

We have reanalyzed the samples obtained by [3] with the new perspective of temporal metatranscriptomics to unravel the dynamics of the rhizosphere microbiome after two acute perturbations: the *Verticillium* infection and the mechanic damage to the roots. This time, we included data from 15 days after infection that were not previously analyzed. Our hypothesis was that the dynamics of these two process should be clearly distinguishable. Our ultimate goal was to use the dynamics of the aforementioned processes to provide insight into the complexity of the interactions between the different organisms thriving in the rhizosphere of a tree.

## Results and discussion

### GC content and mapping of the MTS reads

**Fig. 1:**
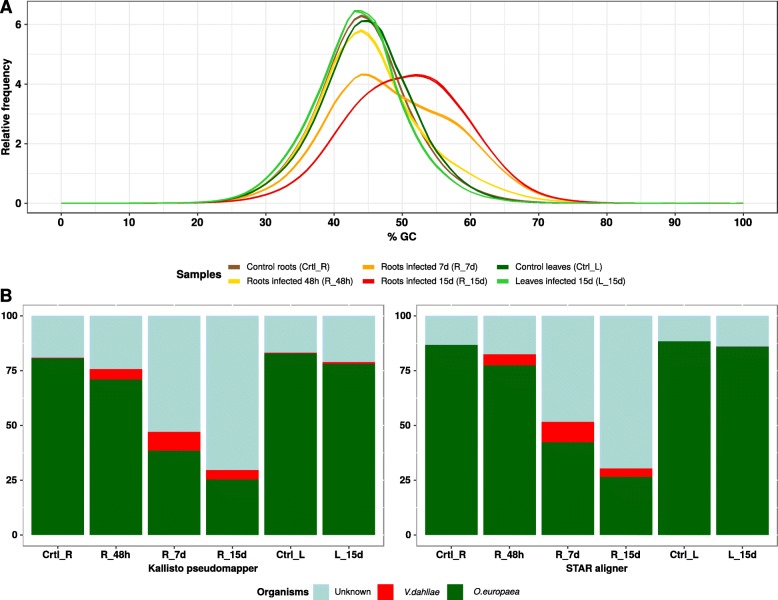
**(A)**: The average per base GC content of the reads. The GC content of the olive genome is roughly 43%. **(B)**: Percentage of reads mapped to the olive (green) and *V.dahliae* (red) genomes, obtained through Kallisto pseudomaper and STAR aligner, respectively. The cyan color corresponds to the proportion of unmapped reads of initially unknown origin

An analysis with FastQC and MultiQC of the sequenced reads showed a notorious change in the per base GC content in the infected root RNA samples that increased with time after the *Verticillium* inoculation (Fig. 1a). The GC content in the sequenced reads evolved from a unimodal distribution peaking at 43% in controls, being coincidental with that of the olive genome, to a pseudo-gaussian distribution reflecting a higher GC content ratio (53%) 15-day after the inoculation. Figure 1b and c show the percentage of reads coming from infected roots mapped either with Kallisto or STAR to the olive genome drastically decreasing along the infection. The number of *Verticillium* reads was very low in all samples, even 15 days after infection. Since infection was performed only through roots, *Verticillium* reads were negligible or even missing in the leaves. So, the per base GC content and the proportion of mapped reads in the leaves from both control and infected plants to the olive genome remained practically constant. All these data taken together confirmed that the infection progressed through the roots, did not reach leaves, and that there was a progressive emergence of other biological organisms during the infection that were displacing the olive tree and the *Verticillium* transcriptomes in terms of mRNA abundance. The species origin of the unmapped reads was unknown, and thus, a metatranscriptomic analysis was required.

Regarding any potential GC content bias, from the different high-throughput techniques for transcripts enrichment in RNA-seq, rRNA depletion methods introduce more biases than poly-A affinity methods [14, 15]. Instead of just a bias towards organisms with low GC content in their genome, it seems that the bias is unimodal, in the sense that both AT-poor and AT-rich fragments are under-represented [16]. While reducing the GC content bias could be essential for differential gene expression analysis [17], that is not our case, as the core of our study was a rank-based dynamics analysis [13]. For all the above reasons, in this study, we did not finally correct any potential bias of the GC content.

### Temporal metatranscriptomic analysis of the root infection process

#### Overall dynamics of the infection

**Fig. 2:**
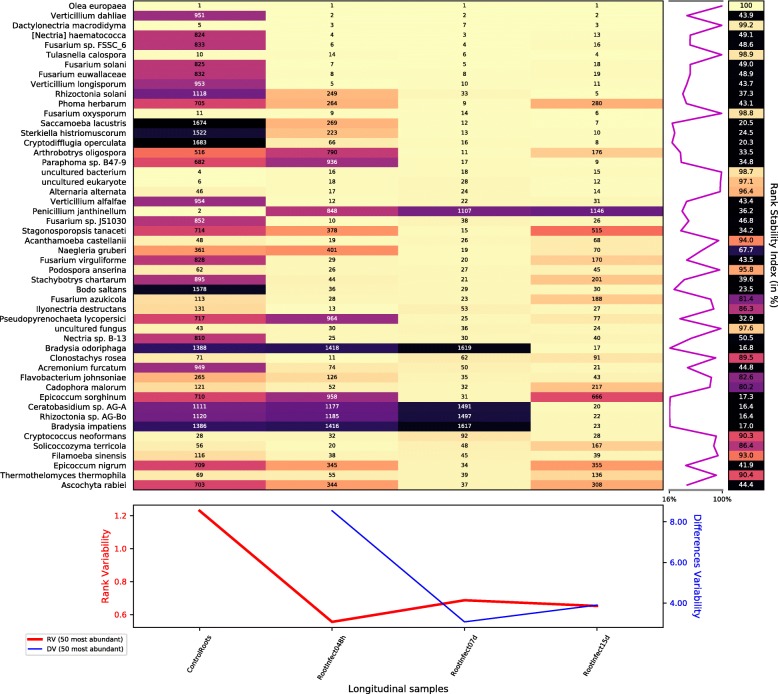
Rank dynamics and stability plot for mapped reads classified at species level during the process of infection with *V. dahliae*. The dynamics of the rank during the process shows the profound impact in the rhizosphere of the olive tree caused by the inoculation with *V. dahliae*. Numbers and colours (using perceptually uniform colormap for easier visualization) show the ranking by the accumulated species abundance in each column. Different rank variability and stability measurements [13] are given. The right panel shows the rank stability throughout species ordered by their overall abundance. The lower panel contains plots of the rank variability (RV) and differences variability (DV) over time

Figure 2 shows how the infection with *V. dahliae* caused a profound impact in the rhizosphere of the olive root. This is shown by comparing the rank dynamics of mapped reads and the stability plot for species of the root control sample (first column) to the roots 48 h after the infection (second column). The boost in the relative frequency of *V. dahliae* reads is the most obvious, but it is not the only change. The same figure shows that other species are taking advantage of the *Verticillium* advance, and some others are suffering an apparent displacement.

The low values in the rank stability index —RSI— [13] column and the extreme fluctuations in the RSI plot indicate that the rhizosphere experienced an intense perturbation with the inoculation of *V. dahliae*, also corroborated by peak values both in rank and differences variability —DV— plots. Thereafter, the rhizosphere was undergoing a transient state as a complex system. The instability is reflected in the lower taxa present in all samples compared to the non-perdurable taxa along the infection (see Additional file 1: Figure S1). Although the entire process is rank-unstable, the analysis of rank dynamics for root species during days two, seven and fifteen days after inoculation (second, third and fourth columns, respectively, of Fig. 2) shows lower sustained values of rank variability, thus indicating that the variation in the populations of organisms after several days of infection is not as intense as after inoculation. The new rank distribution for species may be an early imprint of the form of the Verticillium wilt of olive that will afflict the plant: either the acute or chronic form, also called the ‘apoplexy’ and ‘slow decline’, respectively [2].

**Fig. 3:**
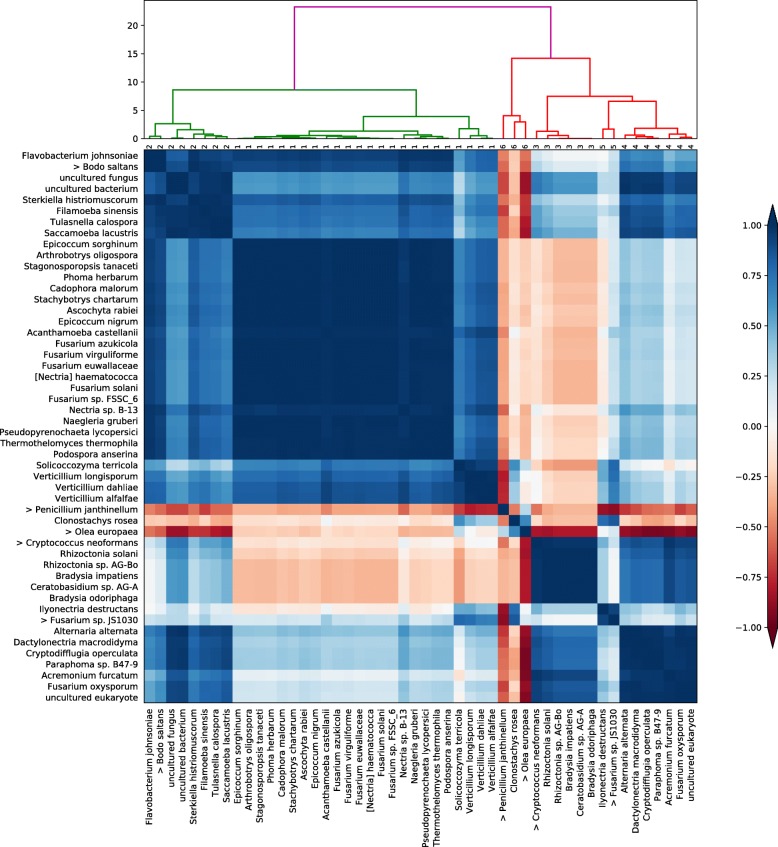
Clustered correlation and dendrogram plot for species during the process of infection with *V. dahliae*. We show the 50 most abundant species ordered by clustering based on the Pearson time correlation matrix (darker shades of blue indicate higher magnitudes of positive correlation, while darker shades of red indicate higher anti-correlation values). With this analysis, six different clusters can be identified (cluster number assignment shown under the dendrogram)

Figure 3, the clustered correlation and dendrogram plot for mapped reads at species level during the infection, shows the evident antagonism between the cluster formed by *Olea europaea*, *Clonostachys rosea*, and *Penicillium janthinellum* (cluster 6), and the cluster containing *Verticillium* spp. (cluster 1). These clusters are located in opposite extremes of the assignments performed by the clustering algorithm, which delimited a total of six main clusters.

**Fig. 4:**
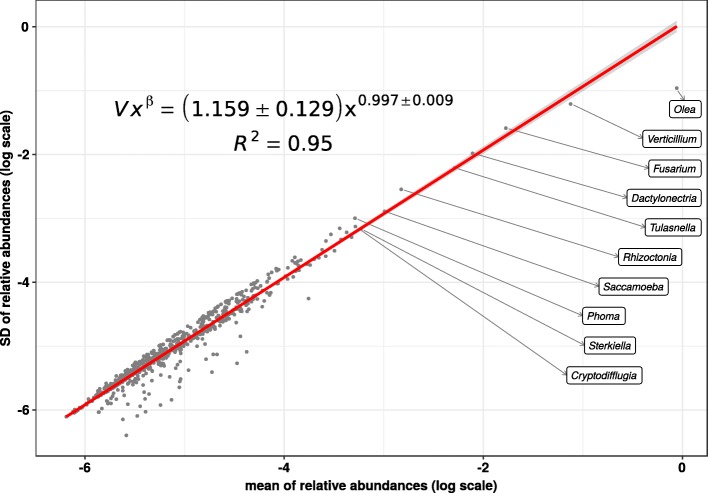
Taylor’s law of the biological system consisting in the metatranscriptome at genus level along the root infectious process. We see that Taylor’s power law spans six orders of magnitude, therefore, it is ubiquitous

Based on our methods for the analysis of microbiota variability and stability [13], we have fit a power-law to std *σ*_*i*_ vs. mean *μ*_*i*_ for the relative abundance of genera during the root infectious process (see Fig. 4). The scaling index *β*∼1 of this Taylor’s law (using the standard deviation as the measurement for dispersion) indicates that the biological system follows the model of an exponential (continuous) or a geometric (discrete) distribution, which is characterized by *β*=1. Since the scaling index *β* contains information regarding the statistical properties of the community structure during infection, that extreme value indicate a peak of overdispersion, thus suggesting an almost uniform volatility for all the range of ginus abundance, which in our case spans six orders of magnitude. On the other hand, the Taylor’s law variability index *V*∼1 is evidence of a very high variability, which implies that the analyzed system is very rank-unstable [18].

Furthermore, Additional file 1: Figure S2 plots Taylor’s law parameter space with data from x-Weighted fits for different taxonomic ranks performed for the dataset of roots olive infection with *V. dahliae* —see [13] for details on the fit. We can see that there is a correlation between *β* and *V* depending on the taxonomic level. We can also see how the no_rank subsample, with no separation by taxonomic level, is located in an intermediate position.

Finally, Additional file 1: Figure S3 shows the Recentrifuge plots of classified reads of fungal MTS at species level for leaves of two different specimens: the control sample and the sample corresponding to 15 days after the *Verticillium* inoculation in the root.

In the following subsections, we present and discuss the evolution during the infection for some significant clades: Amoebae and Cilliates, Fungi, Bacteria, and Nematoda.

#### Amoebae and ciliates

The *Verticillium* infection likely broke trophic network equilibria either directly or indirectly, causing breakage, destructuring of tissues and lysis of cells, thus promoting the grown of opportunistic organisms. In this respect, in Fig. 2 we can see how three protist species (*Saccamoeba lacustris*, *Sterkiella histriomuscorum*, and *Cryptodifflugia operculata*), which were not among the 1500 most frequent species in the root control sample, evolve during the infection to be among the 15 most frequent species found by number of MTS reads assigned. In the rhizosphere, the ubiquitous free-living *Saccamoebae* are living in biofilms and at the interfaces between roots and water [19]. The ciliate *Sterkiella histriomuscorum* (before known as *Oxytricha trifallax*) is a cosmopolitan species in soil, but it is also habitual in limnetic habitats [20, 21]. The amoeba *Cryptodifflugia operculata* is a bacterivore which is also able to prey on larger nematodes thanks to efficient and specialized cooperative hunting [22].

Certain amoeboid protists are pathogenic for the olive. For example, some slime molds of the genus *Didymium* are associated with a severe disease of the olive flower-buds, causing extensive destruction and blockage of flower development [23]. During the infection process, *Didymium* spp. (particularly *D. squamulosum* and *D. iridis*) appeared on day 7, and remained on day 15. In fact, reads belonging to the Myxogastria class, which contains the genus *Didymium*, increased 3.8 times from the control sample to 48h after infection, but they rocketed 10.8 times from 48 h to 7 days after the infection. Taking into account the variation in the absolute number of reads assigned for each sample (see Additional file 1: Figure S4), Myxogastria relative frequency is 2×10^−6^ in the control and about 1×10^−4^ seven days after inoculation. Such a growth is probably due to the increased availability of decaying plant material as a consequence of the appearance of destructive necrotrophic species that took advantage of the *V. dahliae* isolate V937I inoculation, which is an archetype of the highly virulent D pathotype [3].

#### Fungi

**Fig. 5:**
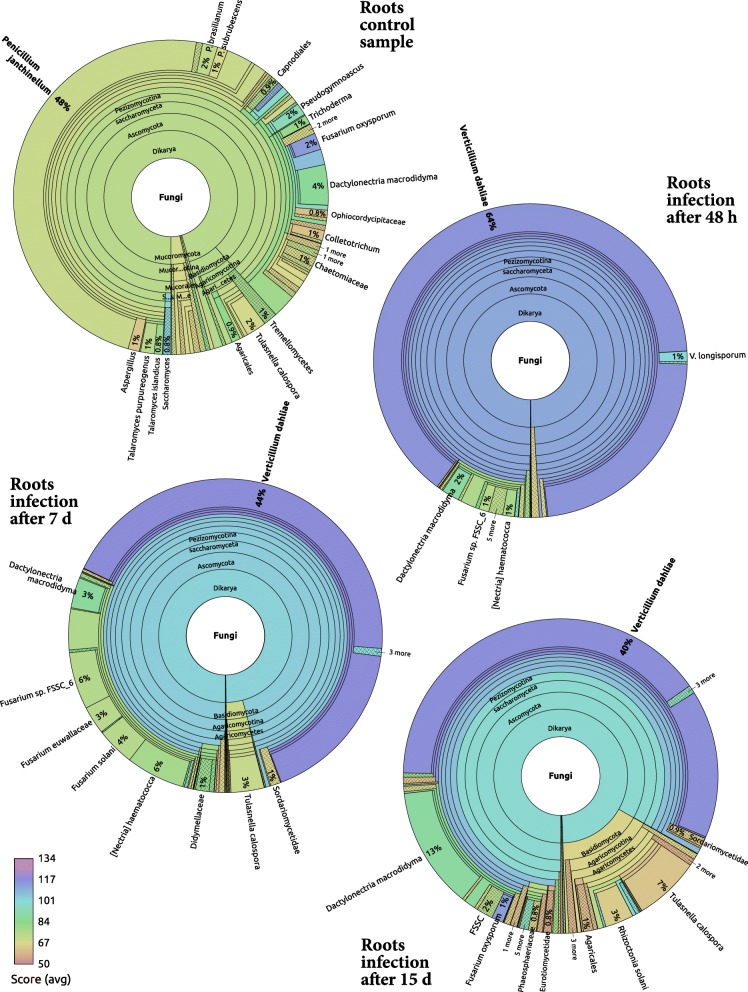
Recentrifuge plots of the evolution of fungal MTS classified reads at species level during the *V. dahliae* infection. The top pie refers to the root control sample, whereas the others apply to the infected roots after 48 h, 7 days, and 15 days, respectively, of the *Verticilium* inoculation. An interactive and dynamic collection of Recentrifuge [24] plots can be accessed via the official project’s webpage at https://www.uv.es/martijm/olea

Figure 5 is a collection of four Recentrifuge plots [24] showing the evolution of fungal MTS reads during the *V. dahliae* infection of the olive root. *Penicillium janthinellum* dominates the root control sample before the infection. *P. janthinellum* is an endophytic fungus which seems to be remedial to plants in the alleviation of heavy metal stress by enhancing the host physiological status. Therefore, it is not by chance that *Olea europaea* and *Penicillium janthinellum* appear clustered together in Fig. 3.

As expected, the frequency of mapped reads indicated that *V. dahliae* became the dominant fungus in the root soon after the inoculation. Its number of reads were the second most frequent just after those pertaining to the olive host (see Figs. 2 and 5). Nevertheless, in the following samples, without losing the second position, its relative frequency started to decrease in favor of other fungi (see Fig. 5) that we review below.

*Dactylonectria macrodidyma* is a fungus that was already present in the control sample, but which benefited from the *V. dahliae* infection since it becomes the third most frequent species in the last temporal point (see Fig. 5) just behind the host and the inoculated fungi (see Fig. 2). *D. macrodidyma* is itself another pathogenic and necrotrophic fungus in crops as it is the causative agent of root rot disease of many herbaceous and woody plants such as grapevine, avocado, cherimola and olive [25], some of them fruit trees whose top exploiters are Spain and Chile [26]. *Tulasnella calospora* is another similar case to *D. macrodidyma* since it was present in the control sample but ended up as the fourth most frequent species in the time series of the infection. *T. calospora* has been recently studied as a mycorrhizal fungal symbiont of orchids [27], but in our case, it seemed somehow to take advantage of the *V. dahliae* infection probably due to the destructuration, destruction, or lysis of tissues and cells. In fact, species of Tulasnellaceae have been described to be both symbionts and saprotrophs simultaneously [28].

One week after the inoculation, reads assigned to the so named *Fusarium solani* species complex (FSSC) represent a fifth of all the fungal reads (see Fig. 5). *Nectria haematococca* and its asexual counterpart, *Fusarium solani*, are the most relevant species in this complex. While researchers in Spain have reported that *F. solani* is only weakly pathogenic on olive [29], this fungus has caused fatal wilt of *Olea europaea* in Nepal [30].

Additionally, the infected samples also contain *Fusarium euwallaceae*, a genealogically exclusive lineage of fungi within Clade 3 of the FSSC discovered as a fungal symbiont of *Euwallacea* sp., an invasive ambrosia beetle that causes serious damage to more than 20 species of olive tree [31]. This taxon, with average score below the paired-ended read half value (100) may represent another close species in the FSSC.

Another frequent *Fusarium* fungi in the studied samples, *Fusarium oxysporum*, is the causal agent of the Fusarium wilt in many different plants, including tomato, chickpea, and others [32], but it is considered only slightly pathogenic for olive in Spain [29]. In fact, it is present in the control root samples and keeps a notable rank throughout the infectious process, reaching its maximum 15 days after the inoculation, where it has advanced to the sixth position of all species (see Fig. 2). Generally speaking, *Fusarium oxysporum* is one of those cases in which a debate is open as to whether this fungus is considered a biotroph, a hemibiotroph, or a necrotroph, able to kill plant tissue quickly and thereafter feeding saprotrophically on the dead remains [33–36].

The fungi *Rhizoctonia solani*, R. sp. AG-Bo, and *Ceratobasidium* sp. AG-A belongs to the same cluster (number three in Fig. 3). As we can see in Fig. 2, these fungi presented a very low frequency of mapped reads during the time series except in the last sample corresponding to 15 days after the inoculation with *V. dahliae*. *Rhizoctonia solani* is a soil-borne plant pathogen that has been related to rotten roots in olives [29]. Both *Rhizoctonia* and *Ceratobasidium* genera belong to the family Ceratobasidiaceae of saprotrophic and cosmopolitan fungi which could be facultative plant pathogens with a wide host range [36].

#### Bacteria

**Fig. 6:**
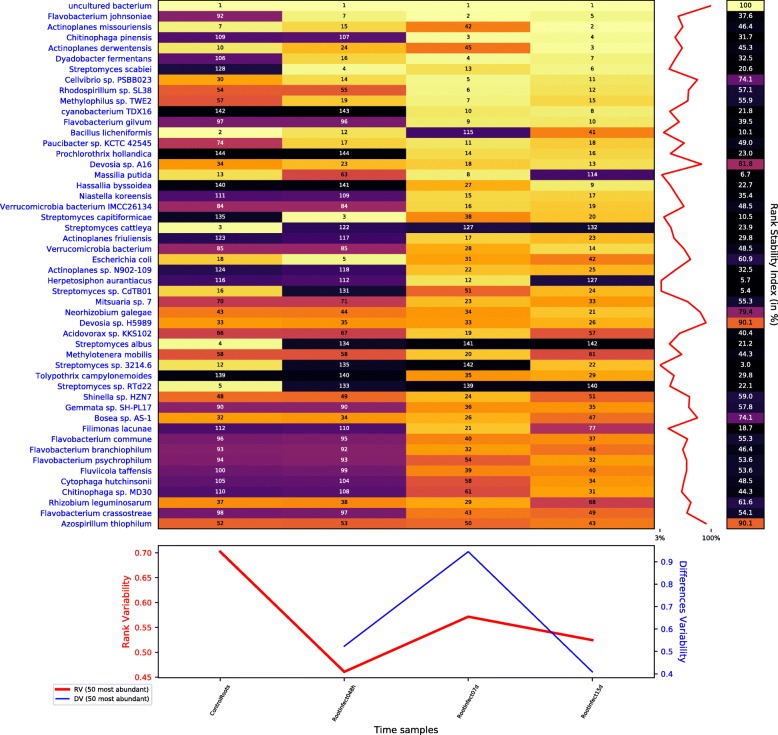
Rank dynamics and stability plot for bacterial species during the process of infection with *V. dahliae*. Numbers and colours (using perceptually uniform colormap for easier visualization) show the ranking by the accumulated species abundance in each column. Different rank variability and stability measurements [13] are given. The right panel shows the rank stability throughout species ordered by their overall abundance. The lower panel contains plots of the rank variability over time

The bacterial content of the samples was severely depleted because the mRNA was isolated using poly-A columns [3], and it is hence, biased. Despite that limitation, the overall dynamics of the bacterial community may still be outlined for the infection. Figure 6 shows the rank dynamics and stability plot for bacterial species. The main difference with Fig. 2, the overall rank dynamics and stability plot for species and dominated by fungi, is the position of the DV [13] peak. In the latter case (general), the maximum is on the second sample —48 h after the infection, while in the former case (bacterial) it appears over the third sample —one week after the inoculation. That means that the effects of the infection reached the bacterial community with some delay compared to the whole species population. The fact that the minimum in DV is on the third sampling time for the whole population but on the fourth sampling time for bacteria supports the existence of such delay.

In Fig. 6, the RSI shows low values compatible with the perturbation introduced in the bacterial community with the inoculation of *V. dahliae*. Intriguingly, a couple of *Devosia* species (sp. A16 and sp. H5989) are exceptions to this behavior since they display high RSI of 90% and 82%, respectively.

Other entirely different cases are *Chitinophaga pinensis* and *Flavobacterium johnsoniae*, which were not very common in the first two samples but then moved forward more than 100 rank positions to reach the top 4 and top 5 in the last two sampling times, respectively. Both are soil-borne bacteria that belong to the widespread and diverse Bacteroidetes phylum and are recognized for its capacity to degrade chitin, the main component in the exoskeleton of arthropods and the cell walls of fungi, so that they could be endohyphal bacteria (a kind of endosymbiont) of fungi belonging to the *F. solani* species complex [37]. Another relevant possibility is that those bacteria could have been recruited by the olive tree through root exudates as an indirect plant defense mechanism against the fungal attack [7, 38]. Indeed, chitinolytic bacteria are well-known antagonists of plant pathogenic fungi [39]. It is well known that bacterial endophytes contribute to the survival and protection of both healthy and stressed fruit plants [40]. In fact, the rhizosphere of wild olive is a reservoir of bacterial antagonists of *V. dahliae* showing chitinolytic activity [41]. The dynamics of *C. pinensis* and *F. johnsoniae* shown in Fig. 6 and the dynamics of species belonging to the FSSC demonstrated in Fig. 2 seem compatible with such hypothesis. The protective role of some microbial endophytes are a promising strategy for the control of diverse pathogens in olive trees, such as *Xylella fastidiosa* [42]. In particular, future studies should extend this approach to Verticillium wilt of olive and, in general, conduct research on the potential biocontrol role of the diverse microbial community related to the olive tree rhizosphere.

#### Nematoda

It is remarkable that *Oscheius tipulae* was detected with both a high score and relatively high abundance on the sample of infected root after seven days. It also appears in the specimen of 8 h after root damage and, with lower abundance, 15 days after the infection. *O. tipulae* is one of the most common and cosmopolitan nematode species in soil [43]. While there is no clear relationship between this nematode and the infection dynamics in this study, it is well known that plants under attack are favoured by soilborne mobile predators such as nematodes, which are efficiently attracted by root-emitted compounds [38]

Although with low frequency and modest score, the presence of *Heterodera* exclusively in samples corresponding to 7 and 15 days after the inoculation with *Verticillium* into roots (see Additional file 1: Figure S5) was of biological significance. *Heretodera* spp. are characterized by their narrow host range, *H. mediterranea* is a well-known plant-parasitic nematode (PPN) associated with the olive tree, especially in nurseries, with reported cases in Spain [44]. Other PPN such as *Meloidogynidae incognita* and *Pratylenchidae vulnus* (absent from the samples of this study) have been associated with *V. dahliae* synergistic co-infections to olives since it seems that, with the indirect root damages that they inflict on the trees, these nematodes act as the spearhead of other pathogenic soil-borne microorganisms like *Verticillium*. Interestingly, Castillo and coauthors suggested that *Heretodera* and *Verticillium* may cooperate synergistically in the Verticillium wilt infection to yield both more widespread and more serious harm to the crop [45]. Our results point precisely in that direction. Finally, with low score, *Bursaphelenchus* also appears in Additional file 1: Figure S5. The genus *Bursaphelenchus* involves a group of predominantly obligate mycophagous nematodes [46]. Generally, *Bursaphelenchus* nematodes feed on fungi or plant cells by using stylets that pierce cell walls thanks to industrially useful *β*-glucosidases degrading enzymes, causing pests in palms and trees [47].

### Temporal metatranscriptomic analysis of the root damage process induced by mechanical injury

**Fig. 7:**
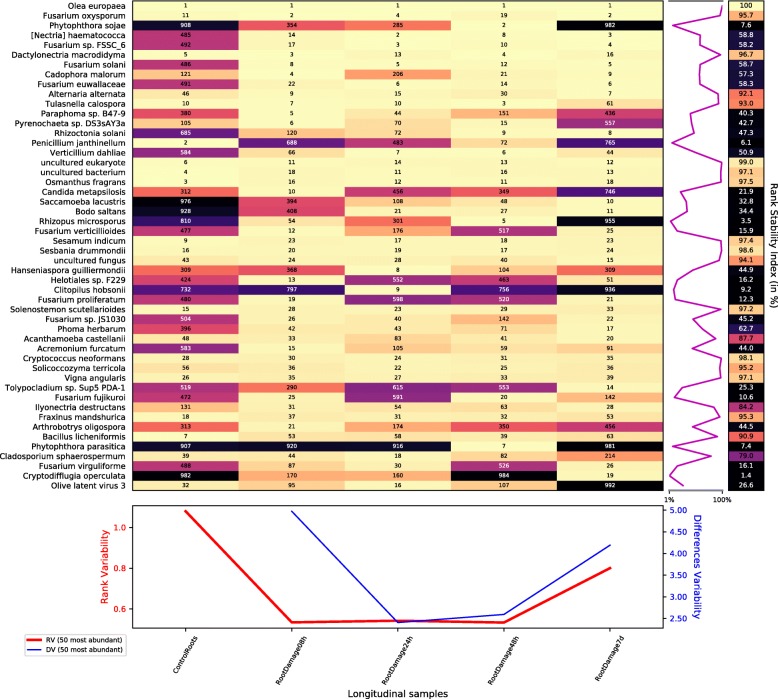
Rank dynamics and stability plot for species during the process after root damage. The dynamics of the rank during the process shows a significative impact in the rhizosphere of the olive. Numbers and colours (using perceptually uniform colormap for easier visualization) show the ranking by the accumulated species abundance in each column. Different rank variability and stability measurements [13] are given. The right panel shows the rank stability throughout species ordered by their overall abundance. The lower panel contains plots of the rank variability over time

Figure 7, the rank dynamics and stability plot for species, shows that the damage of the roots had a substantial effect on the rhizospheric microbiota but less severe than in the case of the infection with *V. dahliae* above. Comparing with Fig. 2, we can see that the rank variability and especially the DV had lower values with the root damage than with the root infection.

However, there were also similarities in the evolution of both datasets despite their different timing. The dynamics of fungi belonging to the FSSC and the drop of *Penicillium janthinellum* abundance after damage are good examples. *P. janthinellum* abundance fell but the slump, being significant, was not as severe as in the infection. *Fusarium* spp. also benefited from the perturbation to the roots, growing as in the infectious case. *Verticillium dahliae* followed this same behavior even when it is in a different correlation cluster than FSSC species, as shows Additional file 1: Figure S6, the clustered correlation and dendrogram plot for species during the process after the root damage.

Nevertheless, other taxa, at the end of the process (7 days), recovered a rank similar to the initial one. That is the case of the plant pathogen *Phytophthora sojae*, which causes root rot of soybean. *P. sojae* had a rank beyond 900 in the control samples, but it reached the second most frequent rank 48 h after damage and returned to minority, tail positions in the last sample. *Rhizopus microsporus*, *Clitopilus hobsonii*, *Hanseniaspora guilliermondii*, and *Arthrobotrys oligospora* behaved similarly, having a final rank close to the initial one after a transient period. In particular, *H. guilliermondii* recovered precisely the same rank at the end (309).

**Fig. 8:**
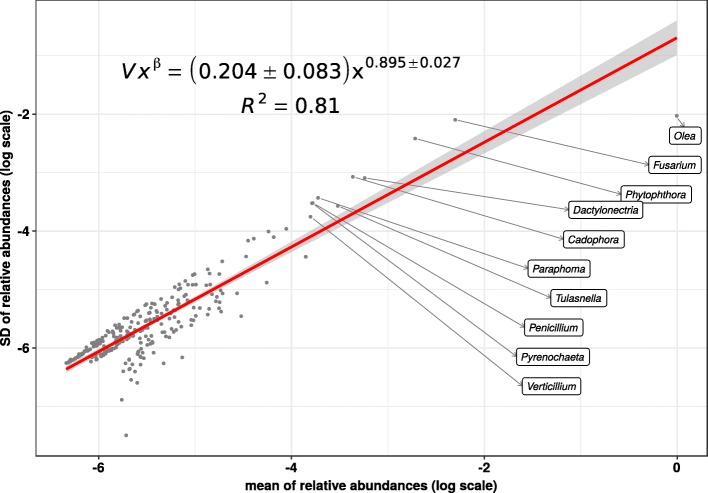
Taylor’s law of the biological system consisting in the metatranscriptome at genus level throughout the root damage process. We see that Taylor’s power law seems to be ubiquitous, spanning in this case more than six orders of magnitude

Figure 8 shows the Taylor’s law fit for the relative abundance of genera throughout the root damage process. Comparing with Fig. 4, we see a lower scaling index *β* and, interestingly, a much lower variability *V*. From a system dynamics perspective [13], these values indicate that the system was more stable after the root damage than after the inoculation with *Verticilium*, thus corroborating the above rank stability results.

Finally, Additional file 1: Figure S7 shows the Recentrifuge plot of MTS classified reads for Dykaria fungi for the sample of leaves 15 days after the root damage. *Candida albicans*, a known human pathogen that has been recently associated with ancient oaks too [48], appears with low frequency but good average confidence.

## Conclusions

**Fig. 9:**
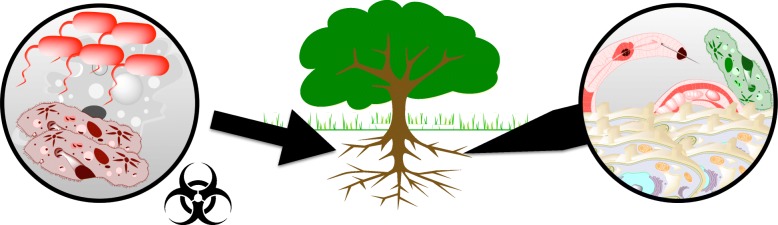
Systems approach to the Verticillium wilt of olive: a complex interaction between complex systems. Polymicrobial community attacks a host community (a host and its symbionts). Our results suggest the relevance of a systems perspective as a generalization of the approach to an infectious process. The tree illustration appearing in this Figure is a public-domain, vectorial image obtained from http://openclipart.org

Our results suggests that the Verticillium wilt of olive is a complex infection process involving more contenders than just *Olea* and *V. dahliae*. This disease, although initially led by *Verticillium*, is driven not by a single species, but by a polymicrobial community acting as a consortium to attack another community formed by the host plant and its natural endophytes, as Fig. 9 represents. This figure depicts how, once *Verticillum* infects the roots, profound changes and alterations of cells and tissues occur. Severe physiological disturbance of the plant host may facilitate infection not only by the new necrotrophic organisms that enter the system, but also by the plant endophytes, which thereafter become harmful. Moreover, the occurrence of opportunistic microorganisms, such as nematodes and amoebas, characterizes the transient process triggered by the *Verticillium* inoculation. Those organisms take advantage not of plant tissues, but from new emerging populations in the rhizosphere. Therefore, Verticillium wilt of olive can be described through a systems biology approach as an intricate biological process in which a complex interaction between several complex systems takes place.

We show that during infection under natural conditions, there is a biological succession of different kinds of parasites (mainly biotrophic and necrotrophic) that could explain (partially, at least) the observed parasitic alternations described in many infective systems. Under our perspective, in order to be able to draw the right conclusions, a careful design of experimental conditions is required to ensure that only the desired parasite thrives. A clear example could be the managing of olive tree saplings and substrates under gnobiotic conditions for plant cultivation.

The temporal metatranscriptomic analysis of the RNA data has allowed us to describe the overall dynamics of the system, as well as to obtain data from amoebae and ciliates, fungi, bacteria, and nematodes. In addition, the temporal analysis of the root damage process induced by mechanical injury has served as a true “dynamic control dataset” of the infection process afflicting the olive rhizosphere.

Our results could also have important implications in relation to the assignment of a specific parasitic species as biotroph, necrotroph or hemibiotroph. *Verticillium*, for example, has been sometimes defined as a biotrophic fungus [49], whereas some other studies define it as hemibiotrophic [50]. Something similar happened with other fungi such as those of the *Fusarium* genus. In particular, our work clearly demonstrates that this kind of assignment cannot be easily performed in an open, natural, and non-sterilized environment, given the enormous complexity of an infection such as that shown in this case, where both biotrophic and necrotrophic species participated simultaneously throughout the process. According to our perspective, a certain fungus should only be recognized as biotroph, hemi-biotroph, or necrotroph when experiments are conducted with plants supposedly raised from both sterilized seeds and plant substrates thus ensuring that only such a particular fungus can grow. In our case, we cannot obtain clear conclusions, since these plants were four month-old potted olives purchased from a commercial and non-controlled nursery [3].

Finally, this study is another example of the usefulness of the draft genomes included in the NCBI WGS database [51], since we have used sequences from draft genomes of olive and fungi to enrich the NCBI nt database. Using the enlarged database, taxonomic classification methods increase their sensitivity [52] and accurately include the information on individual species gathered by the alignment methods.

## Methods

The complete RNA-seq libraries used in this study consisted of duplicated technical replicates (sequencing) of the same biological sample, which were sequenced as unstranded 2×100 paired-ends [3]. They correspond to whole plants heavily infected through roots only with *Verticillium* conidia. Data were downloaded from the NCBI SRA servers with accession numbers provided in the paper of [3] and also in the availability of data section of this document. SRA reads were extracted with the –split-3 legacy option of the fastq-dump NCBI SRA-tool to ensure that paired files were synchronized.

**Pre-analysis** RNA-seq MTS data quality was checked using FastQC v0.11.5 [53] and MultiQC v1.3 [54] software.

**Mapping of reads** The entire MTS library was mapped independently against the genomes of *Olea europaea* (NCBI Reference Sequence: NC_036246.1) and *Verticillium* (NCBI Reference Sequence: NW_009276921.1). That was achieved using both the Kallisto v0.44 pseudomapper in paired mode and using a total of 100 bootstrapping [55] and the RNA-seq STAR v2.7 aligner [56].

**Database preparation** The database used for the Centrifuge program [57] was generated in-house from the complete NCBI nt database (nucleotide sequence database, with entries from all traditional divisions of GenBank, EMBL, and DDBJ) and index databases [51], downloaded in Dec 2017. Through draftGenomes [52], that database was supplemented with all the sequences in the NCBI WGS database [51] belonging to the *Olea* genus and the fungi kingdom. Once generated, the Centrifuge indexed and compressed database weighted more than 135 GB. So far, this is the most massive Centrifuge database we have prepared and used successfully.

**Taxonomic classification** The metatranscriptomic sequences were analyzed with the Centrifuge software package [57] version 1.0.3-beta (Dec 2017), run in parallel within a shared-memory fat node, using 8 threads and peaking half a tebibyte of DRAM.

**Post-analysis** The results generated with Centrifuge were post-processed, analyzed and visualized using Recentrifuge [24], release v0.22.1 or later. Initially, we analyzed both technical replicates separately, but the taxonomic classification results for the replicates were so similar (overall differences in the classification accounting for less than 1%) that we decided to join them in a single sample for each sampling point, thus increasing the sensitivity for minority organisms. In the final analysis, Recentrifuge was run in parallel and with the flags --minscore 50 (MHL set to 50) and -x DYNOMICS to prepare the Recentrifuge quantitative output for further downstream analysis [24]. Finally, we adapted the computational protocol detailed in [58] to perform the temporal metatranscriptomic analysis and produce the plots shown.

## Supplementary information

**Additional file 1** Supplementary Figures.

## Data Availability

The datasets analysed during the current study are available in the NCBI repository with the following accession numbers [3]: SRR1525051, SRR1525052, SRR1524949, SRR1524950, SRR1524951, SRR1524952, SRR1525086, SRR1525087, SRR1525113, SRR1525114) SRR1525231, SRR1525237, SRR1524947, SRR1524948, SRR1525213, SRR1525114, SRR1525224, SRR1525226, SRR1525284, SRR1525285, SRR1525286, SRR1525287, SRR1525415, SRR1525416, SRR1525436, and SRR1525437. Furthermore, an interactive and dynamic collection of plots generated by Recentrifuge [24] during the current study are available in the official project’s webpage at https://www.uv.es/martijm/olea.

## Abbreviations

DV: Differences variability
ENA: European Nucleotide Archive
FSSC: *Fusarium solani* species complex
LLR-model: Log-transformed linear regression model
MHL: Minimum hit length
MTS: metatranscriptome sequencing
PPN: Plant-parasitic nematodes
ROS: Reactive oxygen species
RSI: Rank stability index
RV: Rank variability
WGS: Whose-genome shotgun
