# Supplementary material for: Metatranscriptomic dynamics after Verticillium dahliae infection and root damage in Olea europaea
Source: BMC Plant Biol. 2020 Feb 17;20:79. doi: 10.1186/s12870-019-2185-0 (PMC7027230; doi:10.1186/s12870-019-2185-0)
Supplement: Supplementary file 1 — Additional file 1 Supplementary Figures. [file 12870_2019_2185_MOESM1_ESM.pdf]

Additional Files

Figure S1 — Zero relative frequency (ZRF) histogram for all the taxa of samples during the process of infection with *V. dahliae*

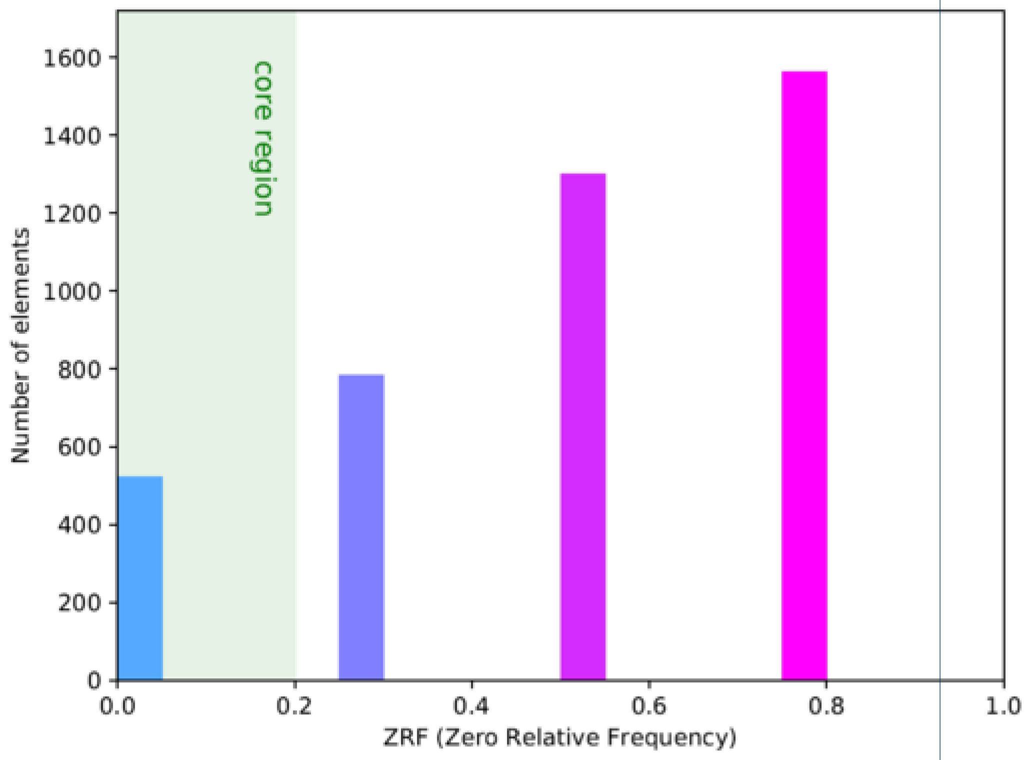

Figures S1 Zero relative frequency (ZRF) histogram for all the taxa of samples during the process of infection with V. dahliae. The 'core' region includes the taxa that are present in all the samples of the study, therefore, the perdurable taxa along the infection process.

Figure S2 — Taylor's law parameters summary plot for x-Weighted fit during the process of infection with *V. dahliae* for various Recentrifuge datasets

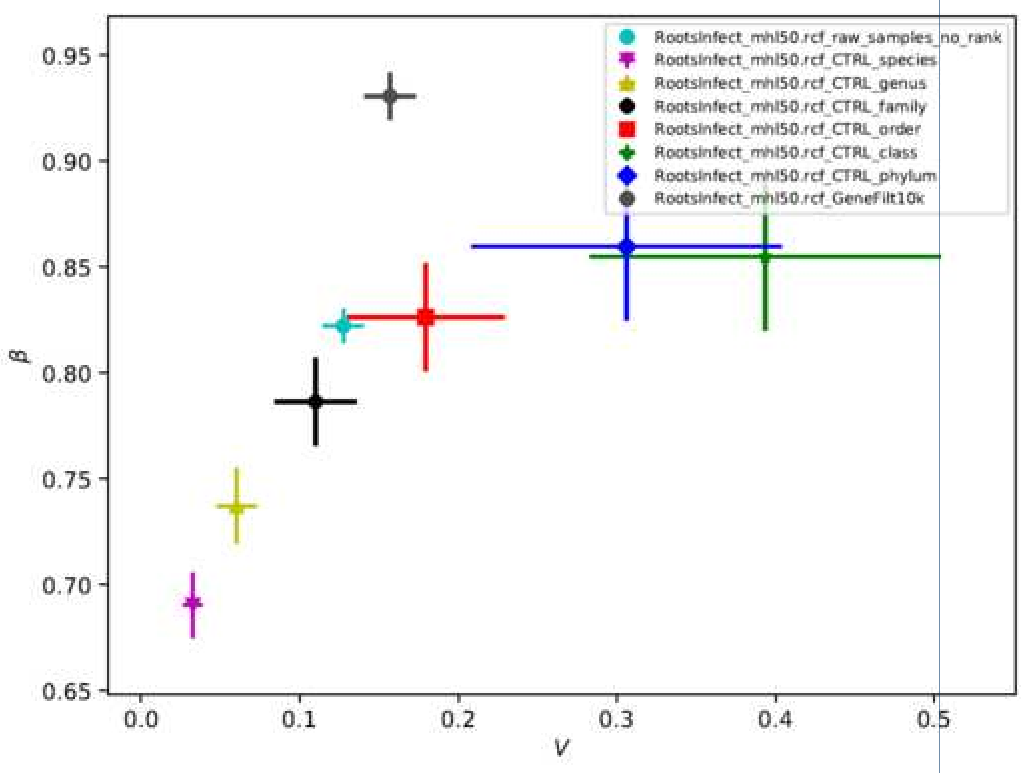

Figures S2 Taylor's law parameters summary plot for x-Weighted fit during the process of infection with V. dahliae for various Recentrifuge datasets. See Martí et al. (2017) for details on the calculations for fitting a x-Weighted model.

Figure S3 — Recentrifuge plots of fungal MTS classified reads at species level for leaves during *V. dahliae* infection

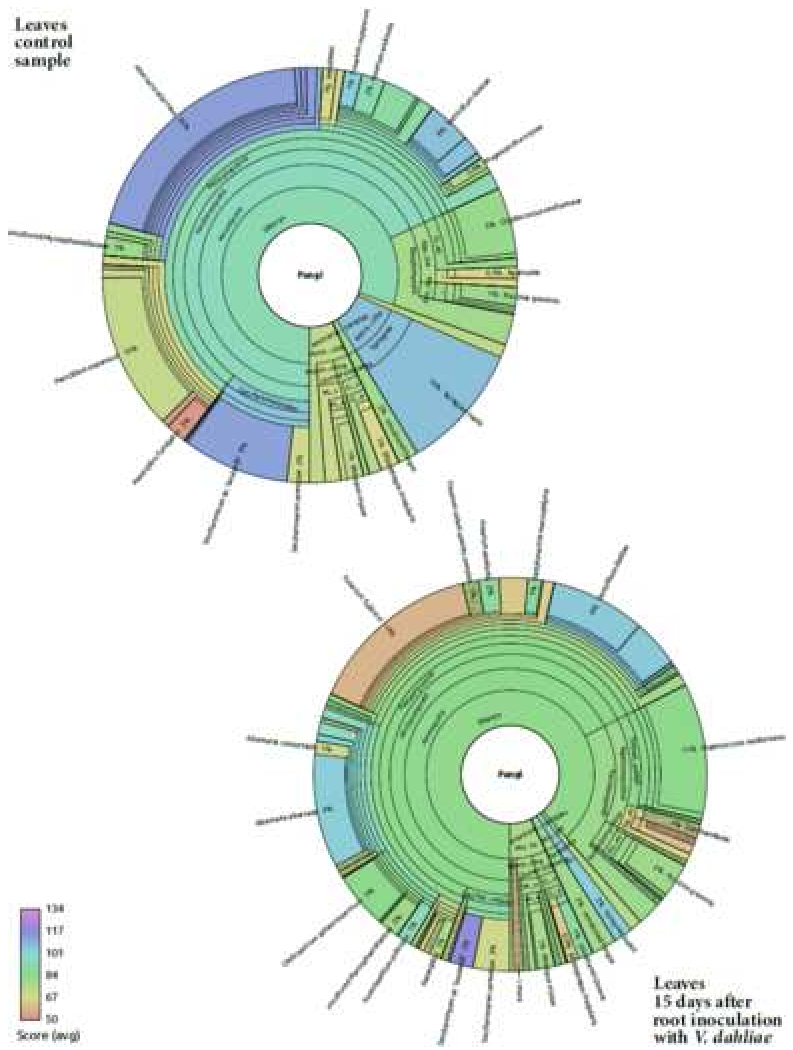

Figures S3 Recentrifuge plots of classified reads of fungal MTS at species level for leaves during V. dahliae infection. The top pie corresponds to the leaves control sample while the bottom chart refers to the sample of leaves 15 days after the *Verticillium* inoculation in the roots.

Figure S4 — Absolute frequency plot for reads of samples during the process of infection with *V. dahliae*

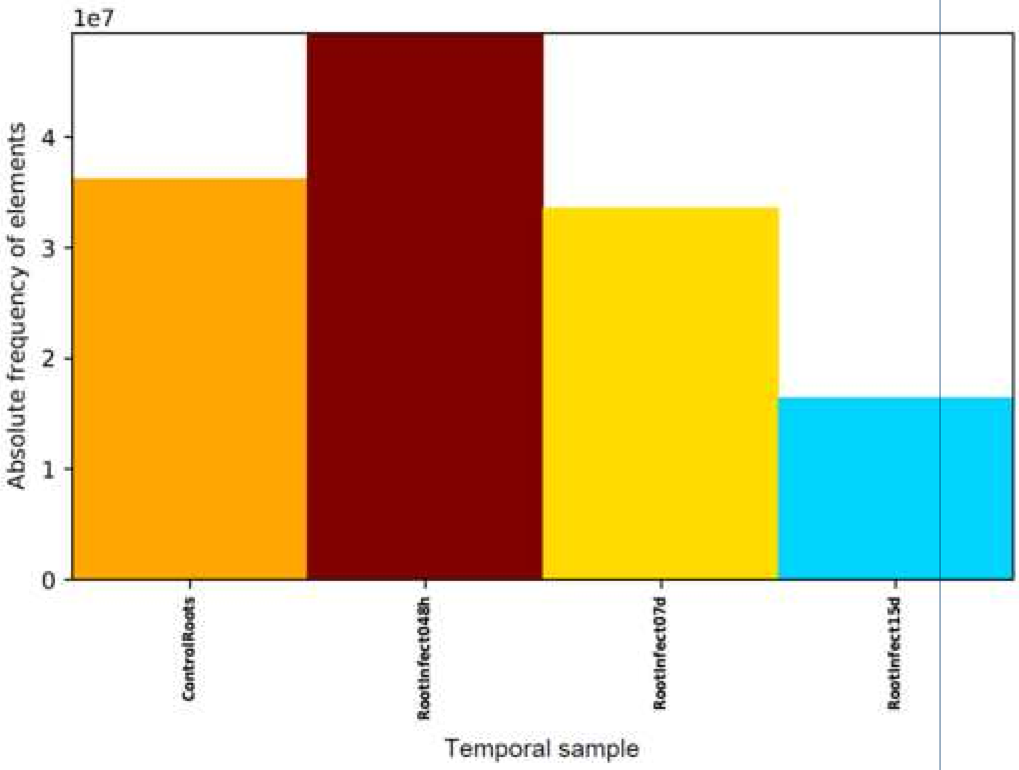

Figures S4 Absolute frequency plot for reads of samples during the process of infection with V. dahliae.

Figure S5 — Recentrifuge plot summarizing the results for suborder Tylenchomorpha 15 days after the inoculation with *V. dahliae*

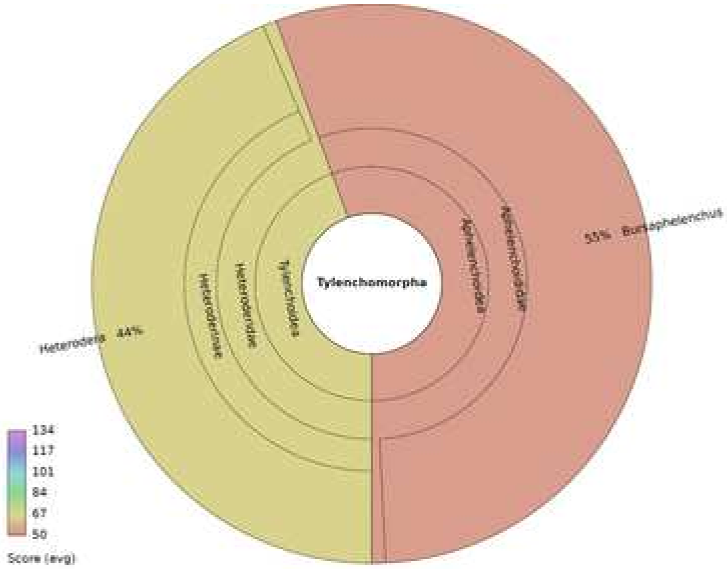

Figures S5 Recentrifuge plot summarizing the results for suborder Tylenchomorpha 15 days after the inoculation with V. dahliae. *Heretodera* and *Bursaphelenchus* genera appear with relatively low scores, but over the cutoff limit of 50.

Figure S6 — Clustered correlation and dendrogram plot for species during the process after *Olea europaea* root damage.

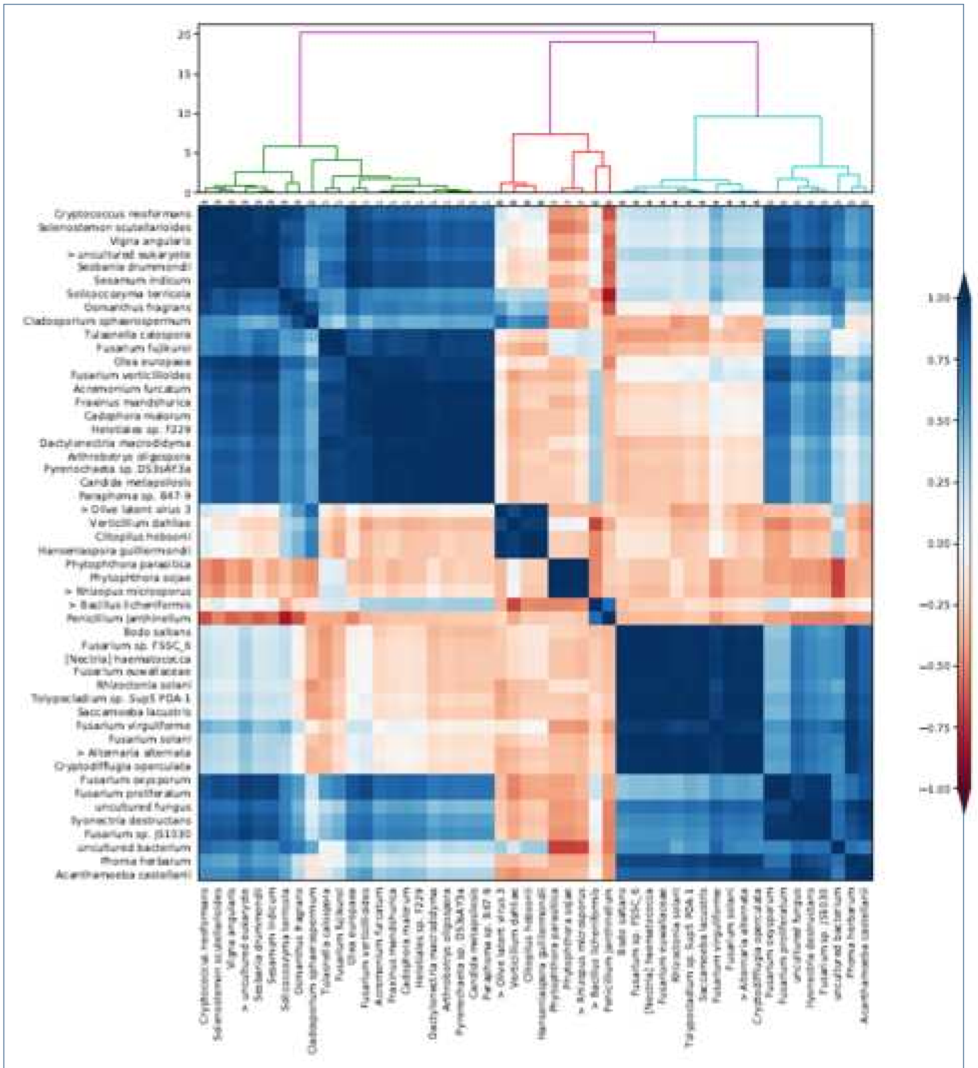

Figures S6 Clustered correlation and dendrogram plot for species during the process after Olea europaea root damage. We show the 50 most abundant species ordered by clustering based on the Pearson time correlation matrix. Eight different clusters (cluster number assignment shown under the dendrogram) can be identified with this analysis.

Figure S7 — Recentrifuge plot of MTS classified reads for Dykaria fungi for the sample of leaves 15 days after the root damage

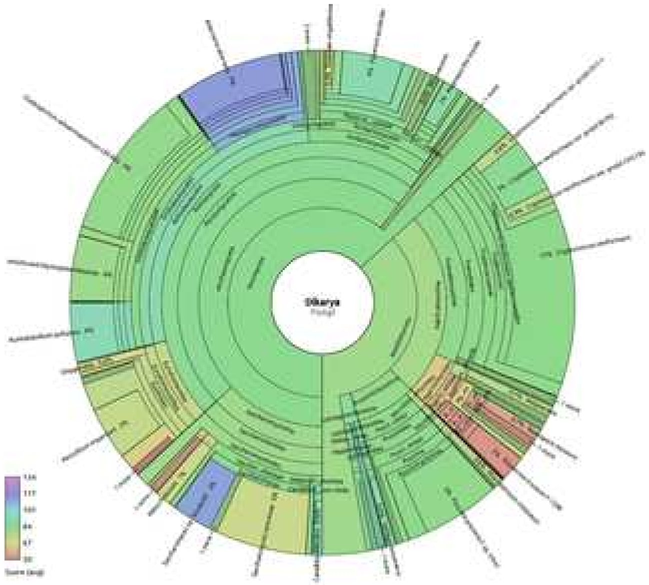

Figures S7 Recentrifuge plot of MTS classified reads for Dykaria fungi for the sample of leaves 15 days after the root damage. In the bottom of the pie, *Candida albicans* appears with a good average score but low frequency.
